# Applications of Vibrational Spectroscopy for Analysis of Connective Tissues

**DOI:** 10.3390/molecules26040922

**Published:** 2021-02-09

**Authors:** William Querido, Shital Kandel, Nancy Pleshko

**Affiliations:** Department of Bioengineering, Temple University, Philadelphia, PA 19122, USA; william.querido@temple.edu (W.Q.); tuf90290@temple.edu (S.K.)

**Keywords:** fourier transform infrared (FTIR), near infrared (NIR), Raman, hyperspectral imaging, fiber optic probes, cartilage, bone

## Abstract

Advances in vibrational spectroscopy have propelled new insights into the molecular composition and structure of biological tissues. In this review, we discuss common modalities and techniques of vibrational spectroscopy, and present key examples to illustrate how they have been applied to enrich the assessment of connective tissues. In particular, we focus on applications of Fourier transform infrared (FTIR), near infrared (NIR) and Raman spectroscopy to assess cartilage and bone properties. We present strengths and limitations of each approach and discuss how the combination of spectrometers with microscopes (hyperspectral imaging) and fiber optic probes have greatly advanced their biomedical applications. We show how these modalities may be used to evaluate virtually any type of sample (ex vivo, in situ or in vivo) and how “spectral fingerprints” can be interpreted to quantify outcomes related to tissue composition and quality. We highlight the unparalleled advantage of vibrational spectroscopy as a label-free and often nondestructive approach to assess properties of the extracellular matrix (ECM) associated with normal, developing, aging, pathological and treated tissues. We believe this review will assist readers not only in better understanding applications of FTIR, NIR and Raman spectroscopy, but also in implementing these approaches for their own research projects.

## 1. Overview

Tissue quality and function are greatly dependent on molecular composition. Advances in analytical methods to assess tissue properties at the molecular level have long provided valuable insights into human health, disease and the effects of therapeutics [1,2,3,4,5,6]. Here, we focus on how vibrational spectroscopy has contributed towards revealing details of the molecular composition and structure of biological tissues. In particular, we highlight the biomedical application of infrared and Raman spectroscopy for the assessment of extracellular matrix (ECM) components of connective tissues. Although we focus on the analysis of cartilage and bone, the approaches described here can be far broader, and may be applied to investigate not only other types of connective tissues (tendon, ligament, dentin), but also a multitude of biological components. For example, vibrational spectroscopy approaches can often be applied to identify, analyze and quantify protein, collagen, proteoglycan (PG), carbohydrates, lipid, mineral, nucleic acids and water, as described in the following sections. Our aim is not only to review some current approaches and prospects, but also to encourage readers who work with connective tissues, but are not necessarily familiar with vibrational spectroscopy, to consider applying these methods to enrich their own research.

To facilitate ease of reading and understanding of the structural organization of this review, we include a Table of Contents that lists sections and subsections (Table 1). The reader will notice that we first describe properties of connective tissues, focusing on cartilage and bone, followed by fundamental concepts of different modalities and techniques of vibrational spectroscopy. We then focus on providing examples of many applications of vibrational spectroscopy for the assessment of cartilage and bone properties, ending with brief concluding remarks on how these approaches have been and can be used in the future to enrich biomedical investigation of tissue composition and quality.

## 2. Connective Tissues

Connective tissues are the most abundant and diverse tissues in the human body, responsible for numerous life-sustaining functions, including support, movement, protection, energy storage and transport of substances. All connective tissues originate from the mesenchyme, and specialize along embryonic development acquiring their own unique properties. The hallmark of most connective tissues is an extensive ECM component, within which the cells are dispersed. The molecular composition and structure of this ECM is essential to carry out the tissue functions [7,8]. Some types of connective tissues are cartilage, bone, tendon, ligament, skin, adipose tissue, fascia, and dentin. Blood can also be considered a connective tissue, due to its plasma matrix, but will not be discussed here. Although the composition varies dependent on the connective tissue type, the ECM often contains collagen fibers, water and glycosaminoglycans (GAG), which play important roles in providing adequate mechanical properties to the tissues [7,8]. Here, we focus on the application of vibrational spectroscopy for the assessment of ECM components of cartilage and bone, but the same or similar approaches may be used to evaluate a variety of connective tissues.

### 2.1. Cartilage

**Figure 1:**
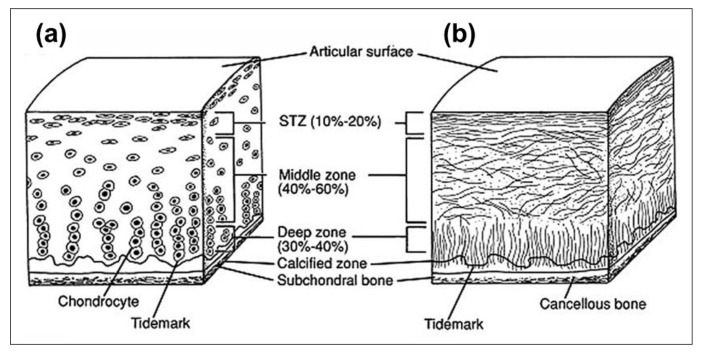
Schematic cross-sectional diagram of a healthy adult articular cartilage: (**a**) cellular organization (**b**) collagen fiber organization in different layers of the cartilage. Image source: Buckwalter et al. [12].

Articular cartilage is a soft tissue that covers the ends of diarthrodial joints. It minimizes the stress on the bone by distributing joint contact forces and reducing the joint surface friction throughout a range of motion. The tissue has a multi-zonal architecture consisting of surface, medial and deep zones, each with a unique composition, and distribution of extracellular matrix components [9,10] (Figure 1). The heterogeneous distribution is related to optimization of load resistance in cartilage [11]. Water is the largest constituent of articular cartilage, accounting for 70% to 80% of its total weight. The major organic components of the extracellular matrix are type II collagen fibrils comprising 60% to 70% of the dry weight, and proteoglycans that comprise 20% to 40% of the dry weight. Chondrocytes are the only cell type found in cartilage and contribute less than 10% of the tissue volume [10,11]. The PG molecules are aggregated around collagen fibers and due to their high negatively charged surface, have a high affinity to water. The relationship between these matrix components varies with the organization within the tissue. The superficial surface layer of cartilage has densely packed type II collagen fibrils that are oriented parallel to the surface, a small amount of PG and a high water content. The middle transitional zone has a lower water content, a higher concentration of PG, and a lower concentration of collagen fibrils that are randomly oriented. The deep zone contains radially oriented collagen fibrils, has the highest concentration of PG, and lowest percentage of water compared to the other zones. As a result of tissue damage or a diseased state such as osteoarthritis (OA), the relationship between these components is disrupted. Since articular cartilage is an avascular and aneural soft tissue, it has a very limited healing potential. Loss of proteoglycans or disruption of the organization is usually the first microscopic evidence of tissue injury, or an early disease process. The increase in water content and a loss of GAG (a building block of PG), are significant changes that occur due to cartilage degradation [11]. These changes are often initiated by the changes in collagen network. The qualitative and quantitative assessment of these changes are critical for early detection of OA and cartilage injuries.

### 2.2. Bone

**Figure 2:**
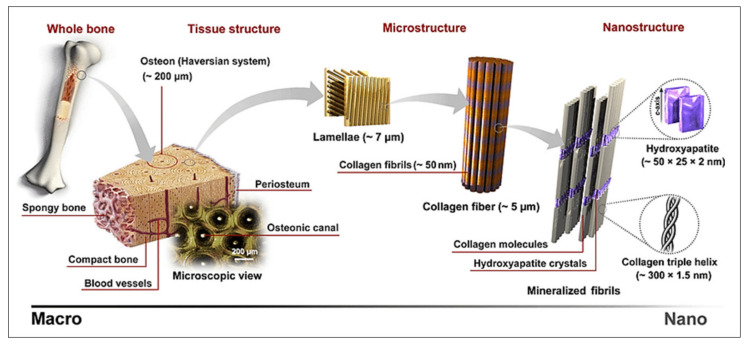
Hierarchical structure of bone from macro to nano scale. The composition and structure of bone tissue at different length scales are essential for bone health and function. Image source: Sadat-Shojai et al. [19].

The primary hallmark of bone tissue is its mineralized ECM, which is comprised of apatite (calcium phosphate) mineral nanocrystals, type I collagen fibrils, water and lesser amounts of a variety of non-collagenous proteins. Bone tissue quality and mechanical integrity are greatly dependent not only on tissue geometry and architecture, but also on the molecular composition and structure of the ECM at different hierarchical levels [13,14,15]. A schematic of the hierarchical structure of bone is shown in Figure 2. At the tissue level, bone is divided into cortical (or compact) and trabecular (or spongy) bone. These two compartments differ in several ways, and the molecular properties of each are directly associated with their function. For example, compact bone is a dense tissue where the ECM is organized into multiple adjacent osteons, which are cylindrical structures formed by concentric tissue lamella surrounding a central vascular channel. In trabecular bone, the tissue forms a mesh of interconnected lamella packets engulfed in bone marrow. Both tissue types play critical roles in bone mechanics and physiology, and assessment of their properties is key to better understanding of bone health and disease. In both cases, the lamellar microstructure of the tissues is formed by arrays of mineralized collagen fibrils displaying ordered alignments and patterns. The mineralization of collagen proceeds through the deposition of mineral nanocrystals within and onto the fibrils, in intimate and organized association with the collagen molecules. At its most fundamental level, the relative amounts, properties and distribution of ECM components are the drivers of bone tissue quality. For example, changes in tissue mineralization degree, collagen and water content, mineral nanocrystal size, and amount of carbonate (CO_3_^2−^) and acid phosphate (HPO_4_^2−^) incorporated in the mineral have been associated with decreased bone strength with aging and diseases, such as in osteoporosis and osteogenesis imperfecta [1,2,16,17,18].

## 3. Vibrational Spectroscopy

Vibrational spectroscopy has been a standard technique in many scientific and industrial endeavors, as a label-free and often nondestructive tool to evaluate composition and molecular structure of a large variety of sample types [1,2,3,16,17,18,20,21,22,23,24,25,26,27,28,29,30,31]. A unique strength of vibrational spectroscopy is its ability to simultaneously assess multiple components based on their inherent molecular constitution. The fundamental basis of vibrational spectroscopy is the interaction of light with the chemical bonds that form a molecule. These chemical bonds are not static, but have periodic vibrations that alter bond length (stretching) and angle (bending). Depending on their atomic and molecular environment, the bonds vibrate with different frequencies, which interact differently with incident light and result in a “spectral fingerprint” comprised of multiple characteristic absorbance bands. The frequency positions, shape and intensities of these bands reflect the presence, properties and amount of different components. Thus, analysis of the spectral data can provide rich insights into the molecular composition of a variety of samples. For example, the intensity of a protein absorbance band correlates to the amount of protein present in the sample, while changes in its position and shape can inform on chemical and structural features of amide (or peptide) bond environments. Moreover, evaluation of the ratio of selected bands can yield important quantitative data from the spectra. For example, measurement of the intensity ratio of a collagen and protein amide band results in assessment of the relative amount of collagen in a sample. Note that spectral processing (for example, obtaining its second derivative) may be necessary to reveal subtle and overlapping peaks, and that the frequency positions of some bands may vary depending on sample type and instrumentation. We recommend the reader keep this in mind while comparing their own spectral data with those from literature and the frequency values shown here in the tables below. This same principle is often applied in different modalities of vibrational spectroscopy to evaluate a variety of tissue components.

### 3.1. Vibrational Spectroscopy Modalities

Vibrational spectroscopy encompasses two well-established modalities, infrared spectroscopy and Raman spectroscopy. Moreover, infrared spectroscopy can be divided into two distinct types, dependent on the frequency range of light that is used: mid-infrared (MIR) (frequency range 400–4000 cm^−1^) and near-infrared (NIR) (frequency range 4000–12,500 cm^−1^). Of note, infrared spectroscopy in the MIR range is now most commonly referred to as Fourier transform infrared (FTIR) spectroscopy, due to the fact that most instruments incorporate an interferometer where a Fourier transform is used to convert the acquired data to the frequency domain. Infrared spectroscopy is based on the absorbance of incident infrared light by the vibrating molecular bonds within the sample, whereas in Raman spectroscopy, an incident monochromatic laser is inelastically scattered upon collision with the bonds. In both cases, the modified light is detected after interaction with the sample, giving rise to a spectrum comprising absorbance or scattering bands characteristic of specific molecular components. Each vibrational spectroscopy modality has strengths and weaknesses, and have to be chosen carefully considering the experimental goals, conditions and sample types, as described below. In some cases, it is interesting to apply different modalities in a complementary fashion to gain a more complete perspective of different components of the same sample.

It is also important to mention that different modalities and techniques of vibrational spectroscopy have differences in complexity and sophistication of instrumentation, sampling and data analysis. The reader may also need to take this into consideration when choosing the optimal approach for their research, as factors such setup availability, sample preparation constrains, and data acquisition time may play defining roles. For example, FTIR spectroscopy analysis of powdered samples can often be easily carried out in many laboratories using widely available equipment and software, whereas analysis of tissue sections often requires more sophisticated spectral imaging systems and data analysis capabilities. Similarly, some Raman spectroscopy approaches may require customized systems or more complex laboratory setups. Throughout the following sections, we describe some particularities involved in each approach; however, we suggest the interested reader to look into more detail protocol papers to gain better knowledge about practical differences in the application of the different modalities and techniques [22,26,32].

#### 3.1.1. Mid Infrared (FTIR) Spectroscopy

**Figure 3:**
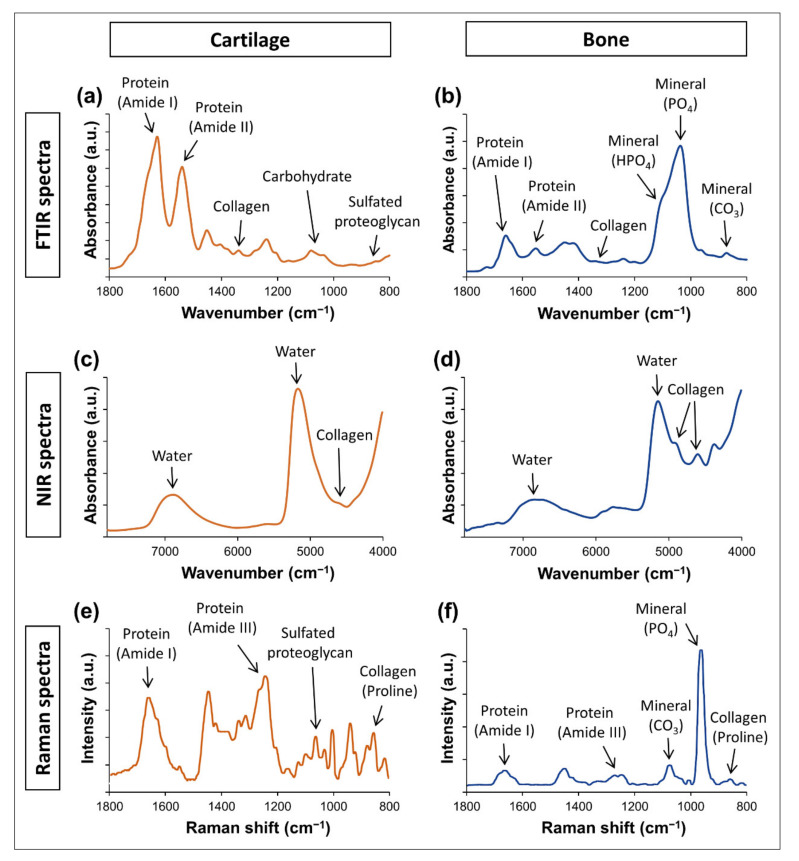
Typical vibrational spectra of cartilage and bone. (**a**) FTIR spectra of cartilage, (**b**) FTIR spectra of bone, (**c**) NIR spectra of cartilage, (**d**) NIR spectra of bone, (**e**) Raman spectra of cartilage, (**f**) Raman spectra of bone.

FTIR spectroscopy is arguably the most widely used vibrational spectroscopy method to evaluate biological tissues. In the MIR range, infrared spectroscopy can assess fundamental stretching and bending vibrations of molecular bonds, resulting in generally intense and precise absorbance bands associated with specific components [1,22]. For example, the frequency of some bands of importance to connective tissue assessment are shown in Table 2. Typical FTIR spectra of cartilage and bone are shown in Figure 3a,b. The reader will notice that absorbance bands of some components have very similar positions, which must be considered before analysis. For example, in the FTIR analysis of a hydrated tissue, it is likely that the absorbance band from water at ~1630 cm^−1^ will overlap heavily with the amide I band from proteins at ~1650 cm^−1^. As a consequence, it is challenging to reliably evaluate the amide I band in hydrated samples, as well as of water in samples that have protein. Similarly, it is not possible to assess carbohydrate bands at 1200–900 cm^−1^ in mineralized bone samples, as this spectral region is often dominated by the phosphate absorbance band from the apatite mineral. It is crucial to understand this limitation in all modalities of spectroscopy, as it can direct the appropriate sample preparation procedure (dehydration, decalcification) to different experimental goals.

It is also important to mention that different sampling modalities exist within FTIR spectroscopy, and having the knowledge to select the right one can be of great value. The most traditional sampling mode is achieved creating a thin pellet comprising a small amount of powdered sample mixed with KBr, which does not absorb infrared light and allows sample spectra acquisition by infrared light transmission thought the pellet. Similarly, fine powdered samples may be dispersed in between two BaF_2_ windows instead of mixed into a KBr pellet, which has the advantage of being nondestructive and requiring less sample preparation. Another very well-established sampling modality of FTIR spectroscopy is attenuated total reflection (ATR), in which spectral data is collected from the sample surface via an evanescent wave. The sample is simply placed in close contact with a crystal, into which the incident infrared light enters and reflects on its internal surface, creating an evanescent wave that interacts with the sample before the reflected light returns to the detector. A clear advantage of ATR is its quick and straightforward data collection procedure, requiring little to no sample preparation and allowing analysis of a variety of sample types, including intact tissues. Its main disadvantage is the very small penetration depth of the evanescent wave, which ranges from ~1–3 µm into the sample, dependent on the wavelength (frequency) being investigated. Additionally, some features of the FTIR spectra may vary depending on the sampling mode [35,36,37,38], and care should be taken when comparing spectra collected using different modalities.

#### 3.1.2. Near Infrared Spectroscopy

The application of NIR spectroscopy for the nondestructive assessment of connective tissues has greatly advanced in the last decade. However, the interpretation of NIR spectra is often not as straightforward as that of FTIR spectra, and understanding the limitations and characteristics of this modality is critical for optimizing its application. Compared to FTIR spectroscopy, the absorbance bands observed in the NIR spectra are typically less intense, broader and less specific, arising from overtones and combinations of fundamental bond vibrations in the MIR range [27,39,40]. The bands are also generally more overlapped, often requiring further spectral processing to reveal underlying features with greater specificity, as described below. Moreover, some components, such as phosphate from the apatite mineral in bone, dentin and other mineralized tissues, are not present in the NIR spectral range. However, absorbance bands from water, protein, collagen, PG and lipids can be identified in the NIR spectra of connective tissues, in the frequency ranges shown in Table 3. Typical NIR spectra of cartilage and bone are shown in Figure 3c,d.

A strength of NIR spectroscopy is the ability to assess tissue water. Analysis of the water absorbances can inform not only on the relative water content, but potentially on the type of molecular environment in which the water molecules exist (free, loosely-bound, tightly-bound), which can also relate to tissue function [50]. However, conversely, water absorbances can overwhelm the spectra when obtained from tissues with high water content or in highly hydrated environments. It is also important to mention that water assessment needs to be done carefully, as sample exposure to the environment can lead to changes in water content in real time due to dehydration or moisture absorbance. This can be of particular relevance when collecting multiple spectra or spectral images that can take long periods of time (sometimes 1–2 h), leaving the sample exposed and susceptible to water content changes during data collection. To overcome this issue, the use of environmentally-controlled chambers may be necessary. For example, Ailavajhala et al. [46] designed a chamber for collection of NIR spectral images of bone. With this chamber, they were able to maintain a controlled environment with optimal conditions to evaluate samples without external influences. To avoid water loss from hydrated samples, the chamber environment was kept at high humidity conditions by pumping it with humidified air. In contrast, to avoid moisture absorbance by dehydrated samples, the chamber was pumped with desiccated air to create a low humidity environment.

Another major strength of NIR spectroscopy is its greater depth of penetration into a sample compared to MIR, due to the higher energy of photons in the NIR range. This makes it possible to assess several millimeters deep into tissues, which is much greater that the few microns possible with FTIR spectroscopy in the MIR range (up to ~10 µm). For example, Padalkar et al. [44] described a wavelength-dependent penetration depth of NIR light into articular cartilage, reaching 1–2 mm in the 4000–5100 cm^−1^ frequency range, 3 mm in the 5100–7000 cm^−1^ range, and 5 mm in the 7000–9000 cm^−1^ range. This advantage greatly expands the applications of NIR spectroscopy to the assessment of a variety of thick or intact tissues with little to no sample preparation and in a nondestructive fashion. Moreover, assessing deeper into the tissue may provide a more complete view of its bulk composition compared to surface analysis. Further yet, considering that NIR light is non-ionizing, NIR spectroscopy may be applied for the analysis of living tissues and organisms, including in humans [51,52,53,54,55,56]. Considerable advances have been made in these applications in the last few years, including in functional NIR imaging [57,58], and tissue imaging [42,46,49,59,60], which shows the potential to provide valuable data on compositional properties associated with tissue quality and health in situ and in vivo.

#### 3.1.3. Raman Spectroscopy

The fundamental concepts underlying Raman spectroscopy are different from those of infrared spectroscopy [26,61,62]. In Raman spectroscopy, an incident monochromatic laser is scattered upon interaction with molecular bond vibrations. Most of the scattered light happens elastically, with no change in the energy and frequency of the light, which is known as Rayleigh scattering. However, a very small amount of the incident light can be scattered inelastically, showing either a loss or a gain in vibrational energy level and a corresponding shift in frequency. This phenomenon is called the Raman effect, described in 1928 by the physicist C. V. Raman, who was awarded the Nobel Prize in Physics in 1930 for his discovery. In the Raman spectrum, the elastically scattered light can be seen as a spectral band with high intensity at the same frequency position as the incident light. This band is called the Rayleigh line. When the incident photons lose energy upon scattering, the Raman bands will be detected in lower frequency positions than the Rayleigh line, which is called a Stokes shift. With gain of energy, the bands are detected in higher frequency positions than the Rayleigh line, showing an anti-Stokes shift. In conventional Raman spectroscopy, only the Stokes bands are typically analyzed, as they are always more intense than their anti-Stokes counterparts.

Several connective tissue components can be identified based on the frequency positions (sometime referred to as Raman shift) of bands observed in the Raman spectrum, as shown in Table 4. Typical Raman spectra of cartilage and bone are shown in Figure 3e,f. An important strength of Raman spectroscopy is that it often requires little to no sample preparation, allowing straightforward data collection from a variety of sample types. Moreover, Raman spectroscopy can have a reasonable depth of penetration into the sample (mms), enabling assessment of thick or intact tissues. Interestingly, in contrast to infrared spectroscopy, water signals are often weak in the Raman spectrum, which avoids interferences from overwhelming water bands when analyzing heavily hydrated tissues. The main limitation of Raman spectroscopy is interferences from laser-induced fluorescence signals that can often obscure scattering bands. This is a particular problem in the case of biological tissues, as the interaction of incident light with some molecules and impurities of the ECM can generate the emission of fluorescence photons with higher efficiency than that of the scattered photons, distorting the Raman spectra. Moreover, even when the autofluorescence interference can be suppressed, the Raman signal from biological tissues is often weak, and signal-to-noise ratio of the spectra may not be optimal. In fact, the approximate signal-to-noise ratio of Raman spectrometers has been described as close to 30–80:1, while FTIR spectrometers can often generate spectra with much greater signal-to-noise ratio, such as close to 1000:1 [18].

An important technical characteristic for the application of Raman spectroscopy is the choice of the incident laser [26]. There is a large variety of monochromatic lasers that can be used in a Raman spectrometer, varying from ultraviolet (UV) to visible to NIR wavelengths. Among the different factors to consider, one of the most important is selecting a laser that can achieve the best compromise between suppression of fluorescence and maximization of Raman bands. In general, lasers with longer wavelength results in better fluorescence suppression, but also requires more acquisition time to compensate for the weaker Raman signals. For biological signals, the most standard laser wavelengths are 785 and 532 nm, although others can also be used depending on the instrumentation. For each tissue type and experimental condition, it is essential to verify in the literature and by trials which incident laser can result in the best spectral quality.

It is also important to mention advances on other Raman-based spectroscopic modalities, in particular coherent anti-Stokes Raman spectroscopy (CARS) and stimulated Raman scattering (SRS) [62]. These modalities require more complex instrumentation, often including multiple laser sources, but can bring important advantages for the assessment of biological tissues. In CARS, multiphoton lasers are used to allow assessing the anti-Stokes signals, which has the important advantage of reducing undesired fluorescence signals from the sample. In SRS, an additional incident light is used to amplify specific molecular bond vibrations and result in more intense Stokes scattering bands. Advances in these modalities have the potential of greatly improving spectral signal while reducing acquisition time.

### 3.2. Advanced Vibrational Spectroscopy Techniques

The application of vibrational spectroscopy has been growing considerably in the past few decades following advancement in instrumentation and optical systems. Two examples of major advances in vibrational spectroscopy are the coupling of spectrometers to imaging systems and fiber optic probes, which enables an important expansion of the biomedical application of these methods. When coupled to imaging systems [1,2,27,31,34,42,46,49,59,60,61,63,65,66,67,68,69,70,71,72,73,74,75,76,77,78,79,80], it is possible to obtain hyperspectral images of tissue sections, in which each micron-sized pixel corresponds to a spatially-defined spectrum. Each hyperspectral image can be comprised of arrays of hundreds to thousands of spectra and allow the analysis of multiple individual components based on the selection of specific intensities or absorbances present in the spectra, presenting an excellent source of information on the amount and distribution of tissue components. Coupled to a fiber optic probe [45,51,52,53,54,55,56,81,82,83,84,85,86,87,88,89,90,91,92], vibrational spectroscopy allows data to be obtained from intact tissues, and living tissues and organisms, in a nondestructive manner, including from human subjects in clinical settings. This represents an important step forward with the potential to translate this method into an advanced diagnostic tool for monitoring tissue development, quality, disease progression and the effect of therapeutics.

#### 3.2.1. Spectral Imaging

**Figure 4:**
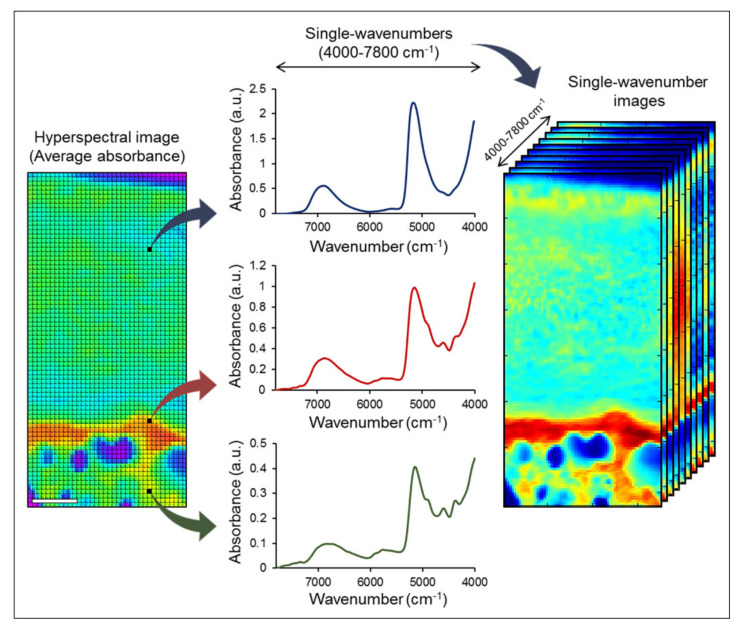
Schematic of hyperspectral image. Each pixel of the spectral image corresponds to an individual spatially-defined spectrum, from which specific features can be extracted to generate multiple compositional maps. The figure illustrates a NIR hyperspectral image of an osteochondral tissue section, in which the intensity of absorbance bands at single-wavenumber frequencies are used to create several images to map the distribution of specific components. Scale bar: 500 μm. Image source: Afara et al. [32].

A major shift in the application of vibrational spectroscopy was the coupling of spectrometers to microscope imaging systems [1,2,27,31,34,42,46,49,59,60,61,63,65,66,67,68,69,70,71,72,73,74,75,76,77,78,79,80]. Both infrared and Raman spectrometers were first coupled to microscopes over 40 years ago, and spectroscopic imaging technology has been advancing greatly ever since. Spectral imaging offers a great advantage over conventional approaches where a single-point spectrum is collected from bulk samples or large sample regions. The main strength of spectral imaging is the ability to collect a matrix of spatially-defined spectra from multiple micron-sized points throughout a region of interest. This data sets a hyperspectral cube with three dimensions (X, Y, Z), where X and Y represent the spatial region of the sample and Z the spectra, containing multiple frequencies (Figure 4). Data from the hyperspectral image cube can be extracted by many approaches, the simplest of which is creation of a two-dimensional map, with X-Y spatial locations and a color scale dictated by a specific spectral feature, such as absorbance band intensity. For example, by assessing the intensity of a protein amide I band in the spectra, we can obtain an image in which regions with greater protein content will be shown as pixels with higher color intensity. A similar approach can be used for bands of other components identified in the spectra (lipid, collagen, PG, etc.). Thus, from one single hyperspectral data set, multiple individual images can be obtained to show the amount and distribution of specific components throughout the same region. This image analysis is often very straightforward, and can be done simply by using the appropriate software to quantify the spectral feature of interest in all the pixels simultaneously, rapidly generating a specific compositional image. Moreover, images of different components can also be overlayed, providing visual representation and quantifiable data on their spatial co-localization.

Hyperspectral images can be obtained by using FTIR, NIR or Raman spectroscopy. Each imaging modality has strengths and weaknesses, and choosing the optimal approach depends on several factors. The first factor is usually determining which modality is better suited to identify the components of interest in each study. As described above, some tissue components can be better assessed by specific modalities. For example, imaging bone mineral would be very difficult to accomplish by using NIR spectral imaging; however, NIR imaging is excellent to assess tissue water content and distribution. The sample thickness is also a major factor. For example, standard transmission or reflectance FTIR imaging requires thin sections, whereas NIR and Raman imaging can often be applied for analysis of thicker samples. Finally, it may be important to consider the desired spatial resolution. The spatial resolution of a hyperspectral image is referred to as its pixel size, which may vary from ~1–50 µm depending on the imaging modality, instrumentation and user-defined acquisition settings. Standard FTIR and NIR microscopes often allow resolutions of ~10–50 µm, while ATR-FTIR and Raman imaging may achieve resolutions down to ~1–2 µm. Ultimately, the choice of spectral imaging modality needs to be done thoughtfully, attending to the experimental goal and conditions of the study at hand.

The spatial resolution is arguably one of the most important aspects of spectral imaging, as it determines the level of compositional detail that will be possible to identify in the final images. In general, the desired spatial resolution value should be smaller than the tissue components of interest to allow a more precise assessment. When the experimental goal of a study is to evaluate very minute tissue components, standard equipment may not be sufficient. Interestingly, more advanced spectral imaging systems have used different strategies to overcome the resolution limits of traditional instrumentation and achieve spatial resolutions at the sub-micron or nanoscale. For example, the combination of Raman spectrometers with confocal microscopes has been widely used to improve spectral imaging resolution to the sub-micron scale [61,73,93]. In a more recent imaging system, a new modality of MIR spectroscopy called optical photothermal infrared (O-PTIR) spectroscopy has been developed to allow sub-micron spectral imaging [94,95,96]. Moreover, this system can combine O-PTIR and Raman spectroscopy to simultaneously image the same sample region, resulting in complementary spectral images using these different modalities [97]. Even further, both infrared and Raman spectrometers have been combined with atomic force microscopes (AFM) to allow obtaining spectral images with spatial resolutions down to ~10 nm [74,98]. These and other advances in spectral imaging technology are clearly shaping a new direction in the field of vibrational spectroscopy, towards allowing imaging the chemical composition of biological tissues in much greater detail.

#### 3.2.2. Fiber Optic Probes

Fiber optic probes have been one of the most powerful tools in modern spectroscopy due to their versatility in both the visible and near infrared frequency ranges, as well as with the use of monochromatic light in Raman probes. They provide flexible solutions for investigation of tissue samples in situ in multiple applications. Numerous clinical applications are possible, including arthroscopic examination of soft connective tissues. Fiber optic probes typically consist of separate illumination and collection optical fibers connected to the light source and the detector, respectively. They merge to the tip of the probe to enable simultaneous sample illumination and light collection. The geometry of the probe tip varies dependent on the optimal coupling to the sample, or on the geometry of the light emission from the probe tip. For example, Cooney et al. studied the sensitivity and sampling volume of beveled fibers compared to other designs. They found that a probe consisting of two beveled fibers is more efficient by at least a factor of 1.5 compared to a dual fiber flat tip when sampling clear liquid [99]. The choice of optical fiber type in a probe depends on the applications and the spectral range. Chalcogenide glasses are widely used in MIR fiber optic probes for the spectral range 900–4000 cm^−1^. In NIR applications, since that spectral region is very sensitive to OH vibrations, low-OH fused silica fibers are widely used. Fused silica fibers are also used in Raman probes due to their very low background signal in the Raman fingerprint region. Santos et al. have shown that silica core-silica clad fibers, with acrylate coating and a black nylon jacket, provided low background signal and good signal quality in in vitro measurements of brain tissue of a 6-month old pig [100]. The versatility of fiber optic probes in cartilage assessment has been shown for in vitro studies of engineered cartilage development [54,55,83,91,101], ex vivo studies of cartilage degradation [81,102], and in vivo arthroscopic evaluation of articular cartilage and subchondral bone [51]. Similarly, Raman spectroscopic probes have also been used to assess articular cartilage quality [103], and have been used to monitor the development of tissue engineered cartilage in vitro [91].

### 3.3. Spectral Data Analysis

#### 3.3.1. Pre-Processing

Spectral data “pre-processing” refers to spectral data processing prior to quantitative analysis, and is usually required to extract reliable information from spectra of physically and chemically inhomogeneous samples such as biological tissues. These techniques improve the robustness and accuracy of the subsequent analysis of the spectra by minimizing effects from background, noise and scattering interferences, and from differences in sample thickness. Various approaches are used to normalize spectra within a range in IR spectroscopy, but spectra may have broad underlying backgrounds [53,104,105]. While ratioing to an appropriate background spectra can reduce much of this effect, first and second order differentiation of the spectra completely removes the background component, attenuating some undesired spectral features, and amplifying resolution of overlapping peaks [106]. Prior to derivative processing, a smoothing filter such as Savitzky-Golay is frequently used to reduce noise. Detrending or deconvolution methods are also used in some spectral analyses to reduce scattering effects, or to decompose superimposed spectral features into individual components. These preprocessing methods can be applied to both IR and Raman spectroscopy data. In Raman spectra, however, removal of cosmic ray artifacts and the effect of fluorescence background needs targeted spectral processing. The cosmic ray artifacts occur when high energy particles hit the sensitive charged couple device (CCD) detectors resulting sharp, intense features in Raman signals. The simplest approach of cosmic ray detection makes use of the temporal randomness of the artifacts and is thus based on the comparison of two consecutive Raman sample measurements [105]. The background fluorescence problem can be overcome by using methods that reduce the fluorescence signal or increase the Raman signal (i.e., photobleaching, photoquenching), removal of fluorophores from the samples, and using UV or NIR lasers that do not stimulate fluorescence [105]. In NIR spectroscopy the most widely used preprocessing techniques are scatter correction techniques (multiplicative scatter correction (MSC), Extended MSC (EMSC) and spectral derivatives, such as Savitzky Golay filter with derivative [107].

#### 3.3.2. Post-Processing

Different univariate and multivariate chemometric methods are used to extract information from the spectral data. A univariate analyses, such as assessment of an absorbance peak intensity or the integrated area of a peaks can provide information on the relative amount of a specific component present in the samples. However, in more complex spectra, including those in the NIR range, overlapping absorbances can be challenging to analyze, and may require a multivariate method. Towards that, principal component analysis (PCA), partial least square (PLS) regression, cluster analysis, and neural network analysis are approaches that have been used to extract information from complex spectra. Such methods are reviewed in detail elsewhere [1,79,108,109], but are described briefly here.

Multivariate data analysis techniques allow analysis of large spectroscopic data sets by reducing the dimensionality and complexity of data so that meaningful information can be extracted. The primary emphasis on multivariate analysis of spectral data is often unsupervised classification of the dataset, as for example has been done using PCA to discriminate healthy and osteoarthritic cartilage [110]. This approach has also been used along with linear discriminant analysis (LDA) for the identification of healthy and OA cartilage in FTIR images of tissue regions and in states of degeneration [111]. Similarly, hierarchical cluster analysis has been used to classify differences in collagen type in hyaline-like cartilage and fibrocartilage-like repair tissue, with collagen II and collagen I mapping respectively [82]. Further, supervised methods such as PLS regression can be employed to extract information regarding compositional changes in healthy and OA cartilage. Such analyses typically require large amounts of calibration data comprised of spectra from tissues with known biochemical composition obtained using gold standard assays. These data are used to create PLS models that are then the basis for prediction of unknown sample composition. Palukuru et al. used NIR spectra obtained from bovine nasal and articular cartilage pellets, and predicted the percentages of collagen and chondroitin sulfate with a PLS model [41]. Similarly, PLS models based on NIR spectra of tissue engineered cartilage were used to predict the tissue composition and mechanical properties of the tissue engineered constructs at different developmental time points [55,56,83]. PLS regression with a variable selection method was used to predict the collagen crosslink concentrations based on FTIR spectral image data from bovine articular cartilage tissue with and without threose treatment to increase cross-link concentrations [112]. In the NIR spectral range, Prakash et al. found PLS regression with variable selection method as an optimal regression approach spectroscopic evaluation of articular cartilage [113]. In addition to these linear analytical methods, nonlinear statistical approaches, including machine learning algorithms such as artificial neural networks (ANN), have been used recently. The Genetic algorithm (GA) approach and least squares version of support vector machines (LS-SVM) methods were implemented by Prakash et al. for NIR assessment of cartilage, but they ultimately found PLS regression to be optimal compared to those methods for prediction of cartilage tissue thickness and mechanical properties [113]. Overall, the use of multivariate analysis methods and machine learning approaches enable extraction of important structural and compositional information from connective tissue samples.

## 4. Application of Vibrational Spectroscopy for Connective Tissue Analysis

### 4.1. Applications for Cartilage Assessment

Vibrational spectroscopic approaches (mid-infrared, near infrared and Raman spectroscopy) provide excellent tools for assessment of articular cartilage. In combination with microscopic imaging, they can be used for the assessment of histologic tissue sections to obtain biochemical composition information at high spatial resolution. These techniques can also be used with fiber optic probes for rapid clinical assessment of cartilage quality. Specific examples are discussed below.

#### 4.1.1. Mid Infrared Spectral Analysis of Cartilage

FTIR spectroscopy and imaging in the MIR range (400–4000 cm^−1^) is a powerful tool to assess normal, regenerating, and degraded cartilage composition. Camacho et al. and Boskey et al. laid down the foundation of IR studies of cartilage with identification of spectral markers of articular cartilage components for semi-quantitative analysis [1,34]. Those studies found that the integrated area of the Amide I absorbance (1590–1720 cm^−1^) correlated with collagen concentration, and the ratio of integrated area of the PG sugar ring (C-O) absorbance (985–1140 cm^−1^) to the amide I absorbance correlated with the quantity of PG. Similarly, the CH_2_ side chain vibration of collagen gives rise to an absorbance peak at 1338 cm^−1^ which is sensitive to the triple helical structure of collagen, and was shown to decrease with the degradation/denaturation of cartilage. The integrated area of this absorbance ratioed to the amide II peak (1490–1580 cm^−1^) was reduced in human osteoarthritic tissues compared to normal tissue [1], indicating changes in the collagen integrity of the articular cartilage. Apart from PG sugar ring absorbances, also known as a carbohydrate peak (985–1140 cm^−1^), the integrated area of an absorbance at 850 cm^−1^, associated with a C-S bending mode from PG molecules, was shown to correlate to PG content in the cartilage [1]. Although some MIR absorbances of cartilage are clearly associated with specific molecules, in some spectral regions processing of data is required to isolate specific peaks. A study by Rieppo et al. found that second derivative spectra were useful to tease out absorbances from collagen and proteoglycans in the 1000–1300 cm^−1^ range of data from bovine cartilage. They performed enzymatic removal of PG and observed that peaks at 1062 cm^−1^ and 1374 cm^−1^ arose from proteoglycans specifically [24]. Additionally, polarized infrared light can be used with FTIR imaging to reveal the zonal distribution of differently oriented collagen fibers based on the ratio of the amide I to amide II absorbances [69]. The information in these foundational studies of native cartilage has subsequently been applied in many areas of cartilage research, including towards understanding of degraded osteoarthritic tissues [102,114,115,116], and characterization of developing engineered constructs [60,117,118,119].

#### 4.1.2. Near Infrared Spectral Analysis of Cartilage

**Figure 5:**
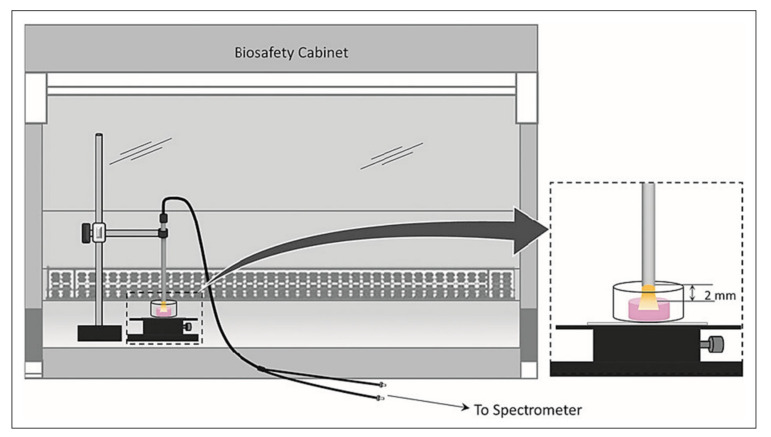
Schematic of the application of NIR fiber optic spectroscopy for assessment of tissue engineered cartilage development. Image source: Kandel et al. [54].

NIR spectra constitutes of combination and overtones of molecular vibrations that typically are observed as broad absorbance bands. Thus, the assignment of absorbance peaks to a specific chemical constituent is challenging, and frequently requires multivariate analyses for assessment of cartilage tissue. However, one excellent aspect of NIR studies is that the NIR radiation has a penetration depth of up to ~1 cm in the spectral regions of interest for connective tissues, and thus fiber optic probes can be used to acquire information from the entire depth of cartilage tissue along with subchondral tissue. Similar to MIR spectroscopy, several peak assignments have been made in NIR spectra of cartilage with absorbance peaks from water and extracellular matrix components. These include the peak at 5200 cm^−1^ associated with free and bound water in cartilage [43], whereas the peak at 7000 cm^−1^ reflects free water in cartilage [43,55]. Palukuru et al. found matrix-associated absorbance peaks in the combination band region (4000–5400 cm^−1^) at 4312 cm^−1^ (PGs), 4610 cm^−1^ (collagen) and 4890 cm^−1^ (collagen) using spectral data from pure component mixtures [41]. In a recent study, Kandel et al. also used pure components and mixtures of articular cartilage components to investigate higher frequency NIR regions using an NIR fiber optic probe. They found the absorbance peaks at 6402, 6556, 6687 cm^−1^ to be sensitive to the collagen content in cartilage samples, and the absorbance peaks at 5778 and 5927 cm^−1^ sensitive to both collagen and PG content in the cartilage [54]. Based on these initial peak assignments and data, hyaluronic acid-based tissue engineered cartilage development was monitored non-destructively with an NIR fiber optic probe [56]. In that study, it was found that the development of tissue engineered cartilage plateaued at week 4 based on the biochemical and mechanical assays, and was also reflected in the PLS model based on the NIR spectra of the constructs. More recently, Kandel et al. found that engineered cartilage construct quality could be longitudinally monitored during development based on the NIR spectra of the constructs using the baseline offset of raw NIR spectra, and PCA [54]. These studies strongly support the use of NIR fiber optic spectroscopy for non-destructive assessment of developing cartilage in in vitro environments (Figure 5).

Several NIR spectroscopic studies have been carried out by the Afara and Toyras groups as well to assess the utility of NIR fiber optics in in vivo arthroscopy studies [120,121]. These were focused on the relationship between NIR spectral data and cartilage biochemical and mechanical properties in normal and disease states. One study showed the application of PLS-R using NIR spectra as a correlate to the swelling characteristics of cartilage based on the PG content across healthy and mechanically degraded cartilage [122]. In another study, NIR spectroscopy combined with machine learning techniques successfully distinguished healthy and diseased cartilage [123]. Another study used PLS modelling techniques to predict the thickness of the healthy and injured cartilage [124,125], and biomechanical, histological and biochemical properties of articular cartilage [125]. PLS-discriminant analysis (PLSDA) based on NIR spectra was also used to investigate mechanical properties of bovine articular cartilage samples [126]. Most recently, the optical properties (absorption and scattering coefficients) of articular cartilage tissue in the visible -NIR region (600 to 2500 nm) were assessed and found to be associated with proteoglycan content and equilibrium moduli in the superficial zone (absorption coefficient), and proteoglycan content and instantaneous and dynamic moduli in the middle and deep zones (scattering coefficient) [127]. Together, these studies strongly support the use of non-destructive NIR fiber optic spectroscopy to evaluate articular cartilage health and disease in both research and clinical settings.

#### 4.1.3. Raman Spectral Analysis of Cartilage

**Figure 6:**
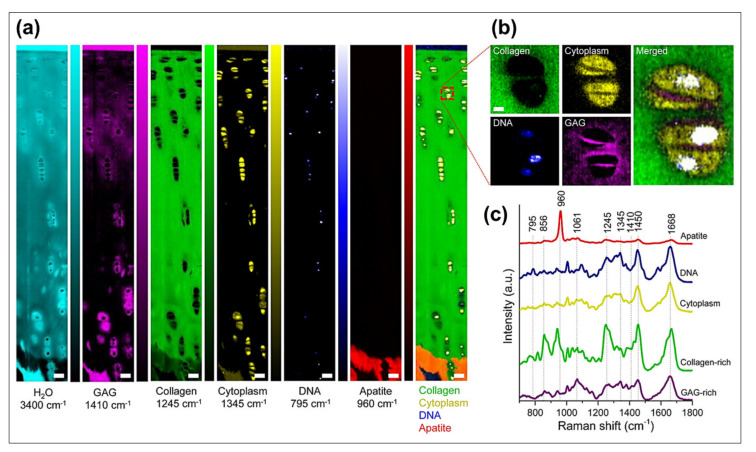
Raman spectroscopic imaging of articular cartilage and the underlying calcified zone. Univariate Raman spectroscopic mapping of articular cartilage based on the band intensity of (**a**) Water (3400 cm^−1^), GAG (1410 cm^−1^), Collagen (1245 cm^−1^), Cytoplasm (1345 cm^−1^), DNA (795 cm^−1^), Apatite (960 cm^−1^) and combined. (**b**) High resolution Raman spectroscopy image of chondrocytes and periceulluar matrix based on collagen, DNA, Cytoplasm, GAG and combined. (**c**) Representative Raman spectra of these articular cartilage components. Image source: Bergholt et al. [67].

Raman spectroscopy is based on the inelastic scattering of the light from the sample when its focused with a monochromatic (laser beam) light. The majority of light scattered (elastic scattering) is filtered out and the weaker inelastic scattering forms a characteristic spectrum of the sample corresponding to the molecular vibrational energies. Raman spectroscopy does not require any sample preparation, and is not affected by the hydration of the sample. Similar to FTIR, Raman spectroscopy can also be combined with microscopy providing a label free Raman microimaging of tissues. Raman spectroscopy and imaging has been applied to numerous tissues including articular cartilage and bone. Raman peak assignments of cartilage components have been well established including: 795 cm^−1^ (DNA), 856 cm^−1^ (proline), 875 cm^−1^ (hydroxyproline; associated with collagen), 1245 cm^−1^ (amide III, N-H of collagen), 1410 cm^−1^ (COO of GAG), 1450 cm^−1^ (CH_2_ deformation of collagen) and 1668 cm^−1^ (amide I, C=O of collagen) [63,67,70]. These peak intensities can be used to visualize the distribution of these components in the tissue (Figure 6). Bergholt et al. investigated the zonal distribution of components using the aforementioned peak assignments. Further, their study also showed the mapping of collagen fiber orientation throughout the depth of the tissue using the Raman peak intensity ratio at 1668 to 943 cm^−1^ [67]. Apart from these univariate or bivariate analyses, multivariate analysis such as PCA can also be used for study of chondrocytes and the extracellular matrix components. Bonifacio et al. demonstrated the use of PCA for mapping of chondrocytes and extracellular matrix along the cartilage using Raman imaging [70]. Similarly, PLS regression models were also used for the semi-quantitative analysis of DNA, Chondroitin sulfate, collagen and non-collagenous proteins as well as relative changes of these components in OA using Raman microscopy [70]. Another study by Albro et al. demonstrated the application of multivariate curve resolution (MCR) to quantitatively measure the concentration distribution of cartilage ECM constituents (GAG, collagen, and water) in native, degraded and engineered cartilage tissues [63].

### 4.2. Applications for Bone Assessment

#### 4.2.1. Mid Infrared Spectral Analysis of Bone

FTIR spectroscopy and imaging have long been a standard approach to assess bone tissue quality. Analysis of the specific absorbance bands present in the FTIR spectra of bone allow investigators to obtain a rich collection of properties related to both mineral and collagen [1,2] (Table 5), providing a powerful approach to reveal underlying molecular and tissue characteristics associated with bone health and function. In particular, FTIR spectroscopy has been a valuable tool to assess bone mineral composition and crystal structure, as several mineral absorbance bands (phosphate, carbonate) are often clearly identifiable in bone spectra. FTIR spectroscopy has been used to assess bone since the 1960s [128,129,130], resulting in the publication of numerous studies that contribute to the better understanding of bone tissue properties in a variety of healthy and disease states, as well as to evaluate the effects of therapeutics on bone components [34,69,71,72,131,132,133,134,135,136,137,138]. This can be generally illustrated by a PubMed search for “FTIR and bone”, which shows over 3000 articles published on this subject, with an exponential increase in the last 20 years. Among these, there are several interesting reviews that summarize outcomes from studies in which FTIR spectroscopy and imaging were applied to assess changes in bone tissue properties associated with aging, diseases and drug treatments [1,4,5,16], including very recent ones [2,139]. In this section, we hope to avoid redundancy by limiting our discussion to some illustrative examples of how FTIR spectroscopy can be applied to assess bone properties. We do, however, encourage the interested reader to look within the vast available literature on this subject for other interesting review papers and for more specific application examples relevant to their own research projects.

**Figure 7:**
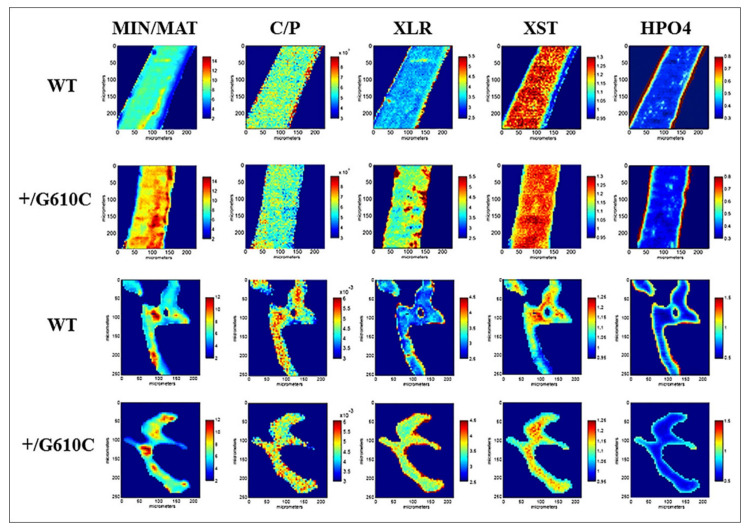
FTIR spectral imaging of cortical (top two panels) and trabecular (bottom two panels) bone from wild type (WT) and +/G610C (OI mouse model) mice. Spectral analysis allowed mapping several compositional parameters of the tissues: mineral relative to matrix ratio (MIN/MAT), carbonate relative to phosphate ratio (C/P), collagen maturity (XLR), mineral crystallinity (XST) and content of HPO_4_^2−^ substitutions in the mineral (HPO4). Image source: Masci et al. [77].

Changes in bone health are often marked by changes in bone ECM at the tissue level, which can contribute to a decrease in bone quality and increase in bone fragility. FTIR spectroscopy has played a key role in revealing changes in the molecular composition and structure of bone ECM underlying aging and diseases, such as osteoporosis, osteogenesis imperfecta (OI) and diabetes. An interesting example of this application was described by Masci et al. [77] studying changes in bone properties caused by α2-chain collagen mutations in a knock-in mouse model of OI. In this study, they not only assessed how this collagen mutation affected bone ECM compared to wild type controls, but also tested if inducing sclerostin inhibition could be a potential treatment to improve bone properties in mutant animals. Interestingly, they applied FTIR spectral imaging with ~7 µm spatial resolution to evaluate both cortical and trabecular bone tissues of tibia and vertebra at different ages, describing as outcomes the quantification of tissue mineralization, carbonate and HPO_4_^2−^ substitutions in the mineral, mineral crystallinity and collagen maturity (Figure 7). Their results showed several differences in the mutant OI mouse model bones depending on tissue type and age, providing new insights into how collagen mutations could affect bone properties underlying OI pathogenesis. Moreover, they showed that sclerostin inhibition improved some bone properties altered in the mutant mice, such as increasing carbonate and decreasing HPO_4_^2−^ substitutions in the mineral. These changes in bone mineral composition are often associated with an increase in tissue maturity, which could have an important contribution in understanding how sclerostin inhibition can improve bone strength.

FTIR spectral imaging has also been applied to evaluate bone tissue heterogeneity, as the spatial variation of bone properties has been emerging as an indicator of bone quality. For example, Wang et al. [80] imaged cortical and trabecular bone tissue from iliac crest biopsies of women with and without bone fracture history, as well as with or without a history of hormone replacement therapy (HRT). As outcomes from their analysis, they obtained pixel intensity histograms reflecting the distribution of tissue mineralization, carbonate substitutions in the mineral, mineral crystallinity and collagen maturity throughout the tissues. Interestingly, they noticed that bone fragility was related not only to the mean values obtained from the images, but also to parameters of their gaussian distributions, such as widths and tails. An important indication from this study was that bone tissue heterogeneity may play an important role driving bone strength, with healthier bones presenting a generally wider spatial variation of their properties, characterizing the presence of both younger and mature bone tissue regions.

Applications of FTIR spectroscopy have greatly advanced our understanding of bone tissue health and function, and also have a potential clinical relevance to aid in the diagnosis of bone diseases. Bone histomorphometry of iliac crest bone biopsies remains a gold standard approach for the tissue level assessment of bone quality in the diagnostic of several metabolic bone disorders, such as osteoporosis, osteomalacia and renal osteodystrophy, as well as to investigate effects of therapeutics [140,141,142]. As described above, FTIR spectral imaging allows visualization and quantification of multiple components and properties of the mineralized bone ECM from a single section without the need for addition of external contrast, offering a clear opportunity to enrich standard histopathological bone assessment. Additionally, minute amounts of biopsy tissue may be quickly assessed by ATR-FTIR spectroscopy to provide bulk measurements of diverse bone properties. Analysis of the FTIR-determined tissue properties, individually or in combination, may be of great value to determine tissue-level bone quality metrics associated with the diagnosis and prognosis of different bone diseases. Although further research is necessary to validate this association, it is possible that FTIR spectroscopy may soon become a standard approach for the histopathological assessment of bone tissue in clinical practice.

Finally, it is also interesting to highlight a few applications by which FTIR spectroscopy revealed new insights into bone tissue ECM composition and organization. For example, FTIR spectral imaging has been a valuable tool for assessment of soft tissue-to-bone interfaces, which are essential to skeletal function. In these studies, compositional images with ~6.25 µm pixel resolution allowed the visualization and quantification of ECM changes across cartilage-to-bone [30] and ligament-to-bone [143,144] junctions. Image analysis showed as outcomes not only distribution mappings of individual ECM components (collagen, PG, and mineral), but also the degree of collagen alignment in the different tissue regions. Interestingly, line scan analyses of the outcome images provided quantitative information on their stepwise variations across the tissue interfaces. For example, using this approach, Khanarian et al. [75] observed that ECM distribution across bovine knee osteochondral interface showed variations dependent on both tissue region and age, providing valuable fundamental information to understand the structure-function relationship of the cartilage-to-bone junction in aging, diseases and drug treatments. In a recent study, another interesting insight gained by the application of FTIR spectroscopy was towards the elucidation of the pathway of bone mineral development. This study showcased a unique strength of FTIR spectroscopy for the identification of a specific absorbance band associated to an amorphous calcium phosphate mineral. By investigating a variety of developing bones, Querido et al. [78] identified the existence of a transient amorphous mineral precursor formed in bone prior to apatite crystallization, which advances our knowledge of bone mineralization and development. It is also important to highlight a recent study by Imbert et al. [74] where bone tissue composition and structure were assessed by AFM-FTIR spectral imaging. Compared to conventional FTIR images with ~6.25 µm pixel resolution, AFM-FTIR images with spatial resolution of 50–100 nm showed a great enhancement in the assessment of tissue properties, identifying not only changes related to tissue maturity but also the alternating pattern of the lamellar structure of individual bone trabecula. Further studies in this field can provide valuable insights into how bone tissue properties at the nanoscale may affect bone quality and strength.

#### 4.2.2. Near Infrared Spectral Analysis of Bone

When compared to FTIR, NIR spectroscopy and imaging has not been as widely applied for bone assessment. This is most likely due to the fact that absorbances from bone mineral are not apparent in the NIR spectra, often rendering this approach not optimal to assess the mineralized bone tissue. However, NIR spectroscopy is highly sensitive to other important components of bone ECM, such as water and proteins, and can thus provide valuable information about key tissue properties. In fact, several studies within the last decade have highlighted the great potential of NIR spectroscopy for assessing bone tissue composition and structure in a variety of sample types and experimental conditions. Although most of the described applications require further development and validation, it is undeniable that NIR spectroscopy holds the potential to become a valuable tool to assess bone tissue properties in the near future.

An important application of NIR spectroscopy is for the nondestructive assessment of subchondral bone tissue within articular joints. Due to the high depth of penetration of the NIR light, it is possible to reach the subchondral bone through the overlying cartilage without damaging the tissue. This approach may be especially relevant to assist the clinical assessment of subchondral bone quality during onset and treatment of degenerative joint diseases. In an interesting early study, Afara et al. [85] used NIR spectroscopy with a fiber optic probe to assess subchondral bone in intact knee joints of rat models with induced-osteoarthritic degeneration. They also analyzed the same samples using micro computed tomography (micro-CT) to obtain standard bone quality measurements. Using multivariate data analysis approaches, they showed that features from the NIR spectra correlated well with the obtained bone mineral density (BMD) and bone volume, highlighting the potential of NIR spectroscopy to assess bone properties. This was later corroborated in a subsequent study with human cadaver knees [86], showing the correlation of NIR spectral features with other micro-CT morphometric properties of subchondral bone, such as plate thickness, trabecular thickness, volume fraction, and structure model index. Further yet, Sarin et al. [51] demonstrated the application of a novel arthroscopic NIR fiber optic probe to assess cartilage and subchondral bone in vivo. The study was performed in juvenile equines, with the probe tip placed inside the knee cavity during surgery. NIR spectra were collected in contact with the cartilage surface, in several spots. The knees were then extracted, and additional NIR spectra were collected ex vivo from the same spots. Properties of the subchondral bone in the corresponding spots were determined by micro-CT. Strengthening the previous findings, they showed that the ex vivo NIR spectra correlated well with bone morphometric properties. Although the correlations obtained with the in vivo NIR spectra were not as reliable, this study represents an important proof-of-concept achievement, which may be improved upon further development of arthroscopic spectral collection approaches.

**Figure 8:**
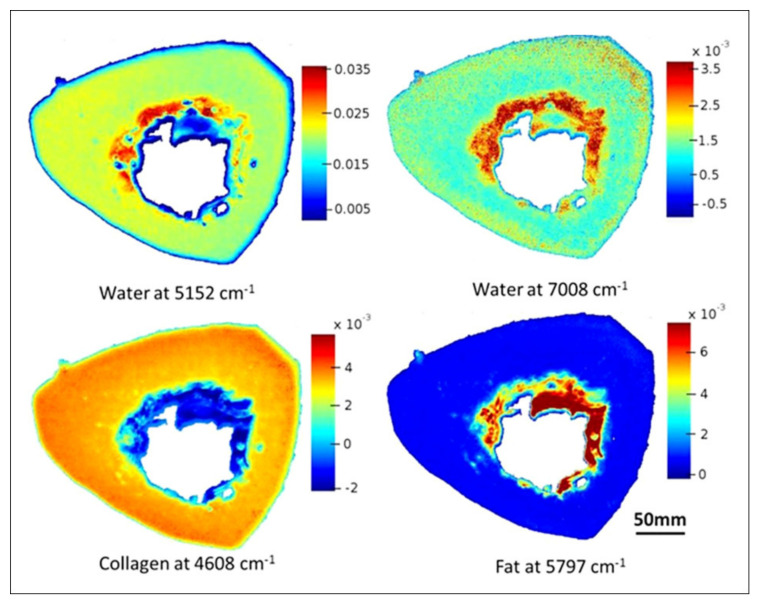
NIR spectral imaging of whole cross-sections of human tibia. The intensities of specific absorbance bands were quantified to produce images showing the distribution of water, collagen and fat within the tissue. Image source: Rajapakse et al. [49].

NIR spectral imaging has also been applied to assess bone composition, providing valuable information on the content and distribution of tissue water and collagen. In an important study, Rajapakse et al. [49] used NIR imaging to evaluate whole cross-sections of cadaveric human tibia. Interestingly, it was possible to image sections with 450 µm in thickness, illustrating the ability of this approach to evaluate thick samples with minimal preparation. Using absorbance bands associated with different ECM components, they were able to obtain images showing the distribution of water and collagen throughout whole bone sections with micron spatial resolution (Figure 8). Additionally, they also used a lipid absorbance band to show the distribution of bone marrow fat at the endosteal surface of the sections. These images represent an important step forward into the application of NIR spectroscopy to assess bone composition at the tissue level. Another interesting finding of this study was that the quantification of water absorbance bands in the NIR spectra correlated well with tissue bound-water content determined by magnetic resonance imaging (MRI), strengthening the application of NIR spectroscopy to reliably assess bone tissue water. Additionally, the intensity of NIR water absorbance bands was later showed to strongly correlate also to gravimetric water content, which is the standard method to quantify water content in tissues. In a subsequent study, Hong et al. [59] further showed a negative correlation between the distribution of bone water and collagen determined by NIR spectral imaging and the porosity index of cortical bone determined by MRI. Moreover, they showed that combining NIR compositional parameters to the porosity index values could potentially contribute to the performance of multivariate regression models to predict bone stiffness. This is an interesting preliminary finding, which encourages research into whether incorporating parameters obtained by NIR spectroscopy into current bone fracture prediction models could improve their accuracy.

A key advantage of the application of NIR spectroscopy is the ability to assess bone tissue water. For example, bone water content can be quantified by assessing the 5184/4608 and 7008/6688 cm^−1^ band intensity ratios, which showed strong correlation (R > 0.90) to standard gravimetric water measurements [46]. Additionally, multivariate analysis by PLS also showed that the NIR spectra of bone can strongly reflect its hydration state [46]. Importantly, this assessment is not limited to content and distribution, but also the molecular environment in which water is located. In bone, water molecules can occupy different compartments, such as within pores (free water), loosely bound to protein and mineral surfaces or tightly bound between collagen chains and within the mineral crystal lattice. Recently, Ailavajhala et al. [47] showed that NIR spectroscopy can be applied to differently assess both loosely and tightly bound water in bone. They used a variety of sample preparation procedures (dehydration, deuteration, demineralization, denaturing) to regulate composition and molecular environments of cortical human bone samples. Using this strategy, they were able to show that the water absorbance bands in the NIR spectra of hydrated bone inform primarily about loosely bound water; however, in dehydrated samples, the remaining water bands are associated to different types of tightly bound water. In these samples, the absorbance band at ~5200 cm^−1^ is associated to a combination of water tightly bound to either collagen or mineral, while the one at ~7000 cm^−1^ is exclusively assigned to water tightly bound to the mineral. In fact, they showed that analysis of this mineral water band in dehydrated samples correlated to tissue mineralization, suggesting that this approach could enable using NIR spectroscopy to indirectly assess bone mineral content and distribution. In summary, these findings highlight the application of NIR spectroscopy to specifically assess different types of bone tissue water, which could have an impact in better understanding the role of water in bone quality.

Finally, it is important to highlight a recent study in which NIR spectroscopy was used in a clinical setting to assess bone quality of individuals with OI, a disease marked by low bone quality and increased fragility. In this study, Shanas et al. [52] used a fiber optic probe to allow collection of NIR spectra noninvasively. The probe tip was simply placed in contact with the individual’s skin on top of the second metacarpal bone. In this region, they showed that the NIR light was able to penetrate through the skin tissue and that a great part of the spectral signals arose from the underlying bone. Interestingly, several NIR spectral parameters associated with water and collagen were found to correlate with standard bone quality measurements obtained by dual-energy X-ray absorptiometry (DXA) and hand X-ray radiographs, as well as with age. The correlated bone measurements included z-score, bone health index (BHI), cortical area and thickness of the metacarpal bone and its relative cortical area (RCA). This preliminary study highlights for the first time the clinical feasibility of the application of NIR spectroscopy for the noninvasive assessment of bone tissue in vivo.

#### 4.2.3. Raman Spectral Analysis of Bone

Analysis of Raman bands results in outcomes mostly equivalent to those obtained by FTIR spectroscopy, such as quantification of tissue mineralization, mineral crystallinity, amount of carbonate substitutions in the mineral, and collagen maturity [2,16] (Table 6). Raman spectroscopy and imaging have been widely applied to investigate bone mineral and matrix composition, as it can be illustrated by numerous publications on the subject [35,145,146,147,148,149,150,151,152], including several comprehensive reviews published in the last decade [2,16,17,18,61,153]. In particular, we can highlight two reviews by Morris and Mandair [18] and Mandair and Morris [17] which focus specifically on the application of Raman spectroscopy to assess bone quality and strength. Additionally, in a very recent review, Taylor and Donnelly [2] present a comprehensive summary of how Raman imaging has been used to evaluate changes in bone tissue composition associated with several diseases, such as osteoporosis, diabetes, osteogenesis imperfecta and renal osteodystrophy. Moreover, the authors show an interesting discussion on the correlation of Raman outcomes with bone tissue mechanical properties at micron- and millimeter-scale, highlighting the importance of tissue compositional properties for bone mechanical integrity. Here, we aim to avoid redundancy by focusing on a few key examples to illustrate applications of Raman spectroscopy and imaging to assess bone properties. However, we encourage the interested reader to look into the previously cited publications for more in-depth examples relevant for their own research. 

An interesting application of Raman spectroscopy was shown by Khalid et al. [145] evaluating changes in mineral and collagen quality in bone samples infected in vitro with *Staphylococcus aureus*. The effects of bacterial growth on bone properties were monitored in using a confocal Raman microscope at different timepoints. The authors obtained several outcomes from the Raman spectra, and found that infection led to an increase in collagen crosslinking, decrease in matrix mineralization, increase in carbonate substitutions in the mineral and decrease in mineral crystallinity. This study shows a promising application of Raman spectroscopy as a tool to aid the diagnostic of osteomyelitis, which could have a great clinical relevance. Moreover, this study illustrates clearly how several properties of bone ECM can be assessed by Raman spectroscopy, describing approaches that can be applied to the analysis of a variety of bone samples in many different research projects. Another characteristic of bone that can be assessed by Raman spectroscopy is tissue-bound water. In an interesting study, Unal et al. [146] identified different water compartments within bone tissue using a custom-made short-wave infrared (SWIR) Raman spectroscopy system and different sample preparation procedures. In particular, they found specific bands associated with collagen-bound water (at ~3220, 3325 and 3453 cm^−1^) and mineral-bound water (at ~3584 cm^−1^). In a following study, Unal and Akkus [147] took these findings further by quantifying the contents of collagen-water and mineral-water based on their peak intensity ratios relative to the band of collagen at ~2949 cm^−1^. Interestingly, they found that these ratios had several significant correlations with bone mechanical properties, including toughness, post-yield toughness, elastic modulus or strength. These studies highlight not only the potential of Raman spectroscopy to assess bone water, but also how water molecules bound to either collagen or mineral may play an important role in contributing to bone health.

**Figure 9:**
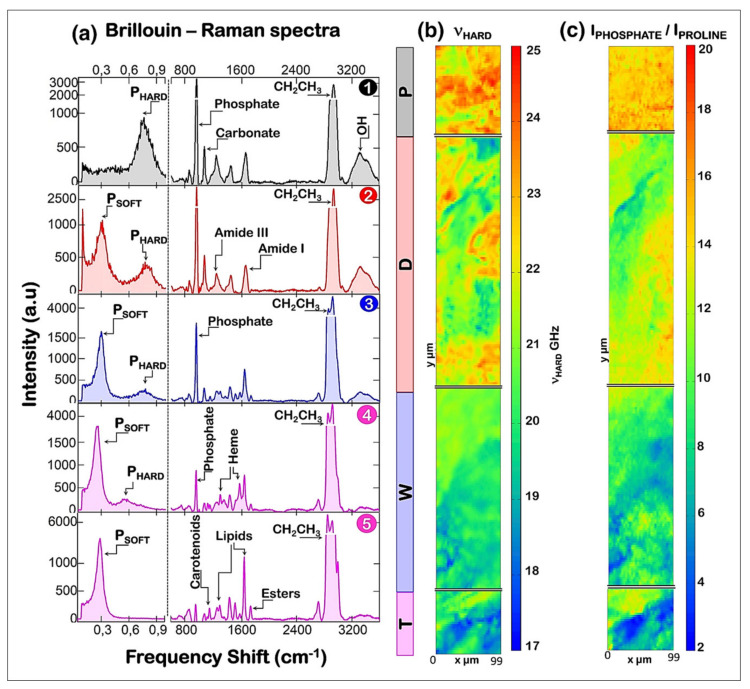
Correlative Brillouin–Raman microspectroscopy. P: periosteal sheath, D: cortical region, W: transition region, and T: trabecular region. From the periosteal surface to the trabecular bone (**a**, top to bottom), note in the Brillouin spectra a decrease in the amount of hard components and increase in soft components. This is followed by a reduction in mineral (phosphate, carbonate) and protein (amide I, amide III) Raman bands and increase in those of lipid and blood (heme). The Brillouin image (**b**) showing the distribution of hard components has a strong spatial correlation with the Raman image (**c**) of the distribution of tissue mineralization (R = 0.78). Image source: Cardinali et al. [154].

Recently, Cardinali et al. [154] described an innovative application of Raman spectral imaging by using correlative Brillouin–Raman microspectroscopy to evaluate the spatial correlation of bone tissue mechanical and compositional properties at the microscale. Briefly, Brillouin spectroscopy is a nondestructive light-scattering technique which can assess the elastic modulus of a tissue, allowing identifying and mapping the distribution of hard and soft components within bone. The authors imaged human diaphyseal femoral cross sections, in regions spanning from the periosteal surface to the endosteal trabecular bone. Brillouin images showed that hard tissue components were primarily found in cortical bone regions, showing a strong spatial correlation with the distribution of tissue mineralization determined by Raman spectral imaging (Figure 9). In contrast, soft tissue components were more present in transition zone and trabecular bone, and were correlated with the distribution of lipids and blood, which arise from the bone marrow. In another very recent study, Kochetkova et al. [155] combined polarized Raman spectroscopy with compression testing to assess the role of collagen alignment on tissue mechanical properties at the microscale. For this study, the authors used a confocal Raman microscope varying the polarization angles of the exciting laser to collect a series of polarized spectra from the same region of interest. Analysis of the amide I/amide III band ratio of the different polarized spectra was shown to inform on the 3D orientation of the mineralized collagen fibrils within the tissue. The authors then used focused ion beam to create micropillars (5 μm in diameter) on the surface of the bone sample for compression analysis using a microindenter. This experimental design allowed them to perform mechanical testing on the same regions where they had collected polarized Raman spectra, making it possible to describe the strong influence of collagen alignment on tissue mechanics at the microscale. Together, these studies illustrate the application of different Raman spectroscopy approaches to reveal insights into how tissue level composition can affect bone mechanical function.

It is also important to mention advances in the application of Raman spectroscopy for in vivo bone assessment. In two interesting studies [65,66], confocal Raman spectroscopy was used to evaluate initial stages of bone mineralization in living zebrafish larvae, revealing the formation of early mineral particles rich in HPO_4_^2−^ inside of cells and close to blood vessels. These findings provide new insights into the pathway of bone mineral development, and in light of very recent advances towards the use of Raman spectroscopy for in vivo imaging of zebrafish tissues [73], more studies in this field are certainly upcoming. Interestingly, in vivo Raman spectroscopy has the potential to go far beyond analysis of zebrafish larvae, including its application for the clinical assessment of bone diseases and healing processes. In the last 10–15 years, the coupling of Raman spectrometers with fibers optic probes has greatly advanced the applications of this technique for non-invasive bone tissue assessment. In particular, an important propeller of this advancement was the development of spatially offset Raman spectroscopy (SORS), in which an array of illumination and collection fibers is strategically arranged on the surface of the tissue to optimize signal detection from multiple positions (spatial offsets) around the region of interest. This configuration allows assessing deeper into the tissue (up to a few cm) [90], which can be a great advantage for the evaluation of bone tissue through skin, fat, muscles and other soft tissues. In fact, SORS has been applied to assess bone properties in vivo transcutaneously in several studies with rodents [156,157,158,159,160] and humans [87,88]. For example, Shu et al. [160] showed that spectral data obtained by SORS transcutaneously from living mice tibiae correlated with standard bone quality metrics, such as areal bone mineral density (aBMD), volumetric bone mineral density (vBMD) and maximum torque (MT). These studies briefly illustrate a potential direction for the application of Raman spectroscopic approaches towards the clinical assessment of bone quality.

## 5. Concluding Remarks

There is a high demand for methodologies that allow a comprehensive and nondestructive assessment of tissue molecular composition and structure. In this review, we discussed some of many ways by which FTIR, NIR and Raman spectroscopy have been applied to meet this demand. The biomedical relevance of these techniques cannot be underappreciated, as they provide a unique approach for the label-free assessment of multiple tissue properties associated with health and disease. In particular, the application of hyperspectral imaging shows an excellent advantage over or complement to current tissue imaging methods, allowing rich information-filled images to be obtained with micron spatial resolution showing the distribution and content of several tissue components (protein, collagen, proteoglycan, mineral, water, lipid) associated with tissue quality and function. With advances in hyperspectral imaging systems, this application can move even further for assessment of tissue composition at the nanoscale. Moreover, the implementation of fiber optic probes for non-invasive data collection shows a promising clinical relevance by allowing evaluation of tissue quality from individuals in vivo, either transcutaneously or during endoscopic procedures. It is important to also highlight that each vibrational spectroscopic modality has strengths and weaknesses, and thus the choice to apply a specific modality and technique should be done with great care and consideration, aiming to achieve specific experimental goals. In summary, we hope this review provides an overall guide to assist with the understanding of how vibrational spectroscopy can be applied in the biomedical field to enrich the evaluation of the molecular composition of connective tissues, and of biological tissues in general.

## Figures and Tables

**Table 1:** Table of Contents.

Section	Page
**1. Overview**	**1**
**2. Connective tissues**	**2**
* 2.1. Cartilage*	*2*
* 2.2. Bone*	*3*
**3. Vibrational spectroscopy**	**4**
* 3.1. Vibrational spectroscopy modalities*	*5*
3.1.1. Mid infrared (FTIR) spectroscopy	5
3.1.2. Near infrared spectroscopy	7
3.1.3. Raman spectroscopy	8
* 3.2. Advanced vibrational spectroscopy techniques*	*9*
3.2.1. Spectral imaging	10
3.2.2. Fiber optic probes	12
* 3.3. Spectral data analysis*	*12*
3.3.1. Pre-processing	12
3.3.2. Post-processing	13
**4. Application of vibrational spectroscopy for connective tissue analysis**	**14**
* 4.1. Applications for cartilage assessment*	*14*
4.1.1. Mid infrared spectral analysis of cartilage	14
4.1.2. Near infrared spectral analysis of cartilage	14
4.1.3. Raman spectral analysis of cartilage	16
* 4.2. Applications for bone assessment*	*17*
4.2.1. Mid infrared spectral analysis of bone	17
4.2.2. Near infrared spectral analysis of bone	19
4.2.3. Raman spectral analysis of bone	22
**5. Concluding remarks**	**25**

**Table 2:** Typical absorbance bands of relevance to the analysis of FTIR spectra of connective tissues. [1,2,22,33,34].

Frequency (cm^−1^)	Tissue Component
1740	Lipid (ester C=O stretching)
1650	Protein (Amide I; peptide C=O stretching)
1630	Water (OH bending)
1550	Protein (Amide II; C–N stretching and N–H bending)
1338	Collagen (side chain CH_2_ vibration)
1115	Mineral (HPO_4_^2−^ stretching)
1060	Carbohydrates (sugar ring C–O stretching)
1030	Mineral (PO_4_ stretching)
875	Mineral (CO_3_^2−^ bending)
856	Sulfated proteoglycan (C–S bending)

**Table 3:** Typical absorbance bands of relevance to the analysis of NIR spectra of connective tissues. [41,42,43,44,45,46,47,48,49].

Frequency (cm^−1^)	Tissue Component
8500	Water (O–H stretching and bending)
7000	Water (O–H stretching)
6688	Protein/collagen (N–H stretching)
5800	Lipid (CH_2_ stretching)
5200	Water (O–H stretching and bending)
4890	Protein/collagen (N–H bending)
4610	Protein/collagen (C–H stretching and deformation)
4310	Proteoglycan (sugar ring vibrations)

**Table 4:** Typical scattering bands of relevance to the analysis of Raman spectra of connective tissues. [2,17,61,63,64].

Frequency (cm^−1^)	Tissue Component
1660	Protein (Amide I; C=O stretching)
1260	Protein (Amide III; C–N stretching and N–H bending)
1070	Mineral (CO_3_^2−^ stretching)
1060	Sulfated proteoglycan (S=O stretching)
960	Mineral (PO_4_ stretching)
850	Collagen (Proline; C–C stretching)

**Table 5:** Typical FTIR spectral analysis approaches to assess bone compositional properties [1,2].

Peak intensity Ratio ^1^ (Peak Frequencies Shown in cm^−1^)	Bone Compositional Property
1030/1650 1030/1550 ^2^	Mineral content (or mineral-to-matrix ratio)
1030/1020 960/1115 ^3^	Mineral crystallinity
875/1030	Mineral carbonate content (or carbonate-to-phosphate ratio)
1115/1030	Mineral HPO_4_^2−^ content
1660/1690	Collagen maturity (or collagen crosslink ratio)

^1^ Appropriate spectral analysis needs to be performed to quantify peak area or intensity, which is often measured as integrated areas or second derivative peak heights. ^2^ This ratio may be a good alternative in hydrated tissues when the Amide I protein band has contributions from water. ^3^ In ATR-FTIR spectra, this ratio was shown to better correlate to bone mineral crystallinity [35].

**Table 6:** Typical Raman spectral analysis approaches to assess bone compositional properties [2,16].

Spectral Parameter (cm^−1^) ^1^	Bone Compositional Property
960/1660 intensity ratio	Mineral content (or mineral-to-matrix ratio)
Full width at half maximum (FWHM) at 960	Mineral crystallinity
1070/960 intensity ratio	Mineral carbonate content (or carbonate-to-phosphate ratio)
1660/1690 intensity ratio	Collagen maturity (or collagen crosslink ratio)

^1^ Adequate spectral analysis needs to be performed to quantify peak intensity, which can often be measured as integrated areas or second derivative peak heights.
